# Synthesis and Evaluation of the Antioxidant Activity of Lipophilic Phenethyl Trifluoroacetate Esters by In Vitro ABTS, DPPH and in Cell-Culture DCF Assays

**DOI:** 10.3390/molecules23010208

**Published:** 2018-01-19

**Authors:** Roberta Bernini, Maurizio Barontini, Valentina Cis, Isabella Carastro, Daniela Tofani, Rosa Anna Chiodo, Paolo Lupattelli, Sandra Incerpi

**Affiliations:** 1 Department of Agricultural and Forestry Sciences (DAFNE), University of Tuscia, Via S. Camillo de Lellis, 01100 Viterbo, Italy; barontinimaurizio@gmail.com (M.B.); r_vale87@libero.it (V.C.); isabella109@alice.it (I.C.); 2 Department of Sciences, University Roma Tre, Viale G. Marconi 446, 00146 Rome, Italy; rosa.anna.chiodo@gmail.com (R.A.C.); sandra.incerpi@uniroma3.it (S.I.); 3 Centro Interdipartimentale di Servizi per la Didattica della Chimica (CIDSiC), University Roma Tre, Via della Vasca Navale 79, 00146 Rome, Italy; 4 Department of Sciences, University of Basilicata, Via dell’Ateneo Lucano 10, 85100 Potenza, Italy; paolo.lupattelli@unibas.it

**Keywords:** phenethyl trifluoroacetate esters, tyrosol trifluoroacetate, hydroxytyrosol trifluoroacetate, lipophilic antioxidants, ABTS assay, DPPH assay, cell culture DCF assay

## Abstract

Polyphenols are natural compounds showing a variety of health-promoting effects. Unfortunately, due to low lipid solubility, their applications in the pharmaceutical, food, and cosmetic industries are limited. With the aim of obtaining novel lipophilic derivatives, the present study reports the synthesis of a series of phenethyl trifluoroacetate esters containing up to two hydroxyl groups in the aromatic ring. Experimental logP values confirmed a greater lipophilicity of the novel compounds compared to the parent compounds. The radical scavenging capacity of all phenethyl trifluoroacetate esters was evaluated by in vitro assays (ABTS, DPPH) and in cultured cells (L6 myoblasts and THP-1 leukemic monocytes) using 2′,7′-dichlorodihydrofluorescein diacetate. These data revealed that the esters showed a good antioxidant effect that was strictly dependent on the grade of hydroxylation of the phenyl ring. The lack of toxicity, evaluated by the MTT assay and proliferation curves, makes these trifluoroacetates attractive derivatives for pharmaceutical, food, and cosmetic applications.

## 1. Introduction

In recent years, antioxidants have become of increasing interest for food, cosmetic, and pharmaceutical applications [1]. In this respect, polyphenols, a large family of compounds found in plants and plant-derived food and beverages, play an important role due to their well-recognized health-promoting effects [2,3]. In particular, they act as protective agents against oxidative attacks by blocking reactive oxygen species (ROS) and reactive nitrogen species (RNS) responsible for oxidative modifications of relevant biomolecules including polyunsaturated fatty acids and nucleic acids [4]. Such structural changes have important implications as they can lead to cell membrane modifications, DNA damage [5], and the development of serious diseases (cancer, diabetes, atherosclerosis, and neurodegenerative and cardiovascular disorders) [6].

Unfortunately, as phenolic compounds show poor lipid solubility, their applications in non-aqueous media (oils, fats, and emulsions) are limited, and their bioavailability in the cellular environment is generally low. These physicochemical properties can be conveniently modified by increasing the lipophilicity of polyphenols, namely introducing a hydrophobic moiety into the molecule without changing the phenolic or catecholic group responsible for the antioxidant activity. As an example, lipophilic polyphenols have been obtained by simple esterification [7,8,9,10,11] or etherification reactions [12] introducing long alkyl chains. As an alternative, in the literature, it is reported that the incorporation of fluorine atoms and trifluoromethyl groups into a molecule increases its lipophilicity and modulates the binding affinity in protein-ligand complexes, thus improving metabolic stability and exerting a relevant effect on the conformation of the molecule [13]. A significant number of drugs and bioactive compounds including phenols and flavonoids are halogenated structures [14,15,16,17]. For these reasons, in medicinal chemistry, fluorination represents a useful strategy to obtain lipophilic derivatives endowed of interesting physicochemical and pharmacodynamic properties [18,19].

Based on these literature data and our expertise on the synthesis of phenolic antioxidants [8,9,10,11,20,21], we obtained a series of novel trifluoroacetate esters from phenethyl alcohols containing up to two phenolic groups in the aromatic ring. Following evaluation of the lipophilicity of all compounds, we tested their antioxidant capacity by in vitro spectrophotometric analyses (ABTS, DPPH assays) and in cultured cells (L6 myoblasts from rat skeletal muscle and THP-1 human leukemic monocytes) after induction of oxidative stress by the radical generator cumene hydroperoxide using the intracellular fluorescent probe 2′,7′-dichlorodihydrofluorescein diacetate (DCF). Using the same cell lines, we also evaluated the capability of the novel compounds to modulate proliferation and cytotoxicity by the MTT assay. Finally, we estimated cell proliferation by cell counts.

## 2. Results and Discussion

### 2.1. Synthesis of Phenethyl Trifluoroacetate Esters

**Figure 1:**
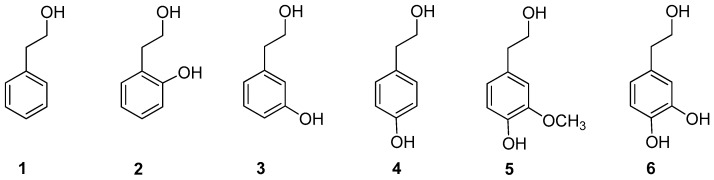
Phenethyl alcohols **1**–**6**.

Biologically active compounds such as phenethyl alcohol **1**, 2-hydroxyphenethyl alcohol **2**, 3-hydroxyphenethyl alcohol **3**, 4-hydroxyphenethyl alcohol (tyrosol) **4**, 4-hydroxy-3-methoxyphenethyl alcohol (homovanillyl alcohol) **5**, and 3,4-dihydroxyphenethyl alcohol (hydroxytyrosol) **6** were used as starting materials (Figure 1).

Phenethyl alcohol **1** is a flavoring molecule found in numerous foods and beverages, in particular beer and wine [22]. It is also an active ingredient of fragrance and antimicrobial preservatives in cosmetic formulations, including make-up and skin care products, shampoos, perfumes, and colognes. The safety of phenethyl alcohol has recently been assessed by the Cosmetic Ingredient Review (CIR) Expert Panel and the Food and Drug Administration (FDA) [23], which included it in the list of substances Generally Recognized As Safe (GRAS) [24]. In addition, it shows antimicrobial [25], antiviral [26] and antitumor effects [27]. The hydroxylated derivatives tyrosol **4**, homovanillyl alcohol **5**, and hydroxytyrosol **6** are low-weight molecular phenols present in extra-virgin olive oil and olive oil by-products [28], well known for their beneficial health effects. Hydroxytyrosol **6** is the most relevant compound, characterized by a potent anticancer and antioxidant activity [29]. It has been widely studied in our laboratories and used for many applications ranging from the synthesis of novel bioactive compounds [30,31] to its inclusion in PVA-based films for active food packaging applications [32,33].

**Scheme 1:**
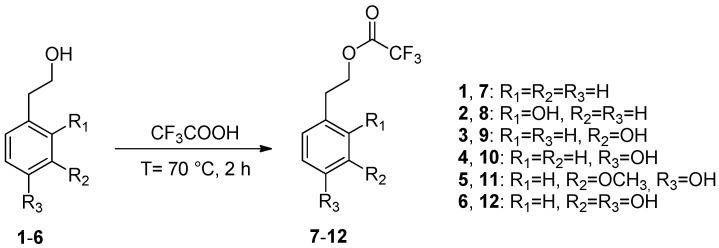
Trifluoroacetylation reaction of phenethyl alcohols **1**–**6**.

In this study, we aimed to increase the lipophilicity of phenethyl alcohols **1**–**6** by derivatizing the alcoholic group with the introduction of a fluorinated moiety. The reactions were performed in the presence of trifluoroacetic acid used as both solvent and reagent at reflux temperature for 2 h. The corresponding trifluoroacetate esters **7**–**12** were obtained in satisfactory to excellent yields and filtered on silica gel to afford pure samples. This synthetic route was original and, as previously observed by us in the carboxymethylation and acetylation reactions of tyrosol and hydroxytyrosol [34], only the alcoholic chain of substrates **1**–**6** was esterified (Scheme 1). However, extending the reaction time to 24 h, trifluoroacetylation also occurred on the phenolic groups.

### 2.2. LogP Determination

Lipophilicity is a relevant property to estimate the bioavailability and dispersion of a molecule in non-aqueous media (oils, fats, and emulsions) and in the cellular environment. Generally, it can be related to logP, a parameter that evaluates the partition of a molecule between two immiscible solvents (water and 1-octanol). The experimental logP values of all compounds were measured by UV-Vis spectrophotometry [35] and are reported in Table 1. As expected, among substrates **1**–**6**, phenethyl alcohol **1** was characterized by the highest lipophilic character, hydroxytyrosol **6** by the lowest value followed by homovanillyl alcohol **5**, while 2-hydroxyphenethyl alcohol **2**, 3-hydroxyphenethyl alcohol **3**, and tyrosol **4** showed similar values. As already reported in the literature [35], the lipophilic character increased from hydroxytyrosol **6** to homovanillyl alcohol **5** and tyrosol **4**.

A similar behavior was observed comparing the logP values of trifluoroacetyl esters **7**–**12**, which in all cases resulted in greater values than those of the parent compounds **1**–**6**. In particular, esters **7** and **8** showed the highest logP values, while compounds **9**–**11** showed comparable values. The increase of the logP value of hydroxytyrosol trifluoroacetate **12** compared to hydroxytyrosol **6** was notable.

### 2.3. ABTS and DPPH Spectrophotometric Assays

**Figure 2:**
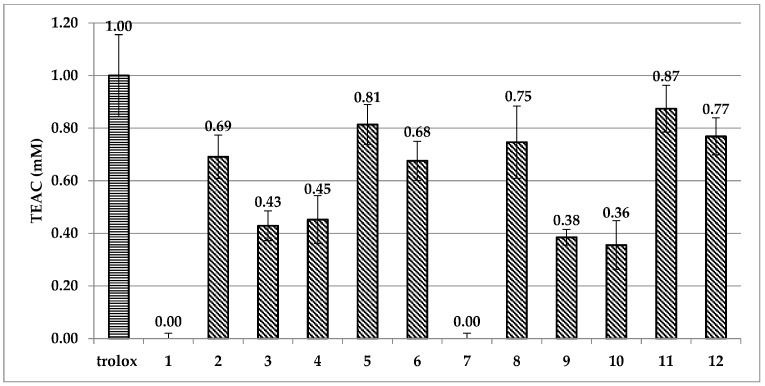
Trolox Equivalent Antioxidant Capacity (TEAC) values, expressed as Trolox mM equivalent, of phenethyl alcohols **1**–**6** and the corresponding trifluoroacetyl esters **7**–**12**. Errors were calculated using Student’s *t* test. Statistical analysis was performed with one-way ANOVA and Bonferroni post-test, *p* < 0.05.

Generally, the aim of in vitro radical scavenging experiments is to provide a preliminary appraisal of the antioxidant capacity of compounds and a premise of the structure/antioxidant activity relationship. As reported in Figure 2, the ABTS assay disclosed a very low antiradical activity of phenethyl alcohol **1** and its trifluoroacetate ester **7**. Phenolic derivatives **2**, **5**, **6** and their corresponding esters **8**, **11**, **12** showed a good antioxidant capacity. Interestingly, in the phenolic derivatives **2**–**4**, the aromatic ring position of the hydroxyl group appears to heavily influence the radical scavenging ability. Indeed, the *ortho* hydroxyl derivative **2** proved to be more antioxidant than the *meta* and *para* isomers **3** and **4**. A similar behavior was observed regarding the trifluoroacetate ester derivative **8**, which showed a higher antioxidant activity than its isomers **9** and **10**. This peculiar effect could be due to the formation of a hydrogen bond between the phenolic proton and the oxygen atom present in the alkyl chain, which may promote the generation of the radical species in accordance to already reported by us [9]. In the catecholic structures, the high hydrogen bond stabilization between the two phenol groups caused the alcohol hydrogen bond stabilization to be insignificant.

In the DPPH assay, IC_50_ values were measured and the anti-radical activities (ARA) were calculated (Table SI1). Comparing with Trolox (IC_50_ 0.221 μM, ARA 4.5 μM^−1^), the low antioxidant capacity of phenethyl derivatives **1**–**4** and **7**–**10** (ARA from 8 × 10^−4^ to 4.5 × 10^−2^ μM^−1^) was confirmed and emphasized by the greater differences in ARA data between these compounds and catechols **5**, **6** and **11**, **12** (ARA from 1.99 to 8.1 μM^−1^). Specifically, TEAC values in the ABTS assay almost doubled going from mono- to dihydroxyl derivatives, whereas ARA values of catechols were at least 40 times higher than monohydroxylated compounds in the DPPH assay. Even though no significant increase of antioxidant capacity was observed in the trifluoroacetate esters, the maintenance of antioxidant activity after esterification was another important affirmation from the in vitro data of ABTS/DPPH experiments (Figure 2, Table SI1).

### 2.4. ROS Determination

The fluorometric DCF assay is a useful technique to evaluate the antioxidant activity of compounds in cells exposed to oxidative stress induced by cumene hydroperoxide. The analyses were carried out in two different cell lines particularly sensitive to radical production: L6 myoblasts from rat skeletal muscle and THP-1 human leukemic monocytes [8,9].

**Figure 3:**
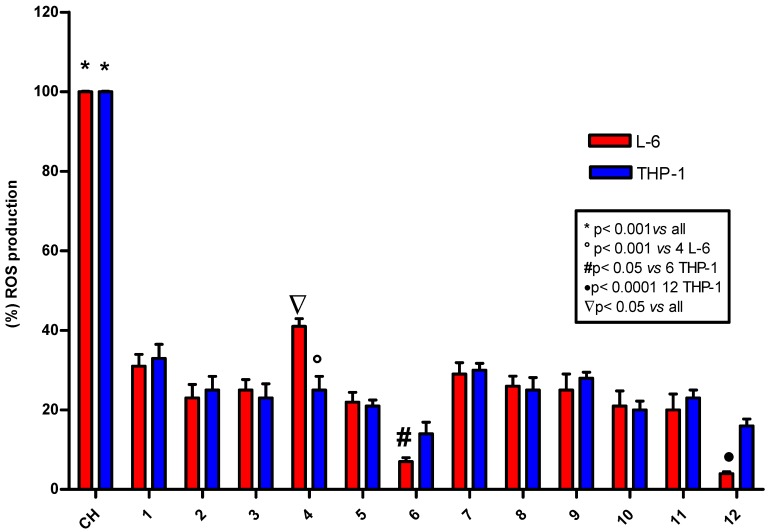
Determination of intracellular ROS in L6 and THP-1 cell lines after stimulation of DCF fluorescence with 200 µM cumene hydroperoxide (CH) alone or in presence of phenethyl alcohols **1**–**6** or the corresponding trifluoroacetate esters **7**–**12** at 10 µM concentration. Data are reported as mean values ± SD of five experiments carried out in triplicate. Statistical analysis performed with one-way ANOVA and Bonferroni post-test, except for **4**, **6**, and **12** where Student’s *t* test was used.

None of the tested compounds gave rise to fluorescence by itself, but all compounds showed an antioxidant activity higher than 70% and between 80 and 90% for hydroxytyrosol **6** and its corresponding ester **12** (Figure 3, Table SI2). The high level of radical scavenging activity of **4**–**6** and their trifluoroacetate esters **10**–**12** was relatively easy to predict considering the general trend observed in the chemical experiments. However, the good antioxidant activity of phenethyl alcohol **1** and its ester **7** was unexpected. As stated earlier, compound **1** has been extensively studied for its antimicrobial, antiviral, and antitumor activity, which is probably related to a physicochemical action on cell membrane permeability. However, to the best of our knowledge, its antioxidant activity has not previously been investigated until now. In this distinctive molecule, the lack of phenolic groups and maintenance of an antioxidant activity supports the notion that the role of antioxidants in cells may be related not only to radical scavenging capacity but also to activation of specific metabolic pathways. Compound **1** is structurally similar to tyrosol **4** and hydroxytyrosol **6**, but also to other simple neurotransmitter monoamines like dopamine. Therefore, the good antioxidant activity of **1** and its trifluoroacetyl ester **7** may be derived from its capacity to interact with specific enzymes or modulators of the antioxidant metabolic pathway, even with just a phenyl ring and alcohol group conveniently located two carbons away from the aromatic [36]. Further biological investigations in this area could be of great interest.

### 2.5. Compound Toxicity and Cell Proliferation

**Figure 4:**
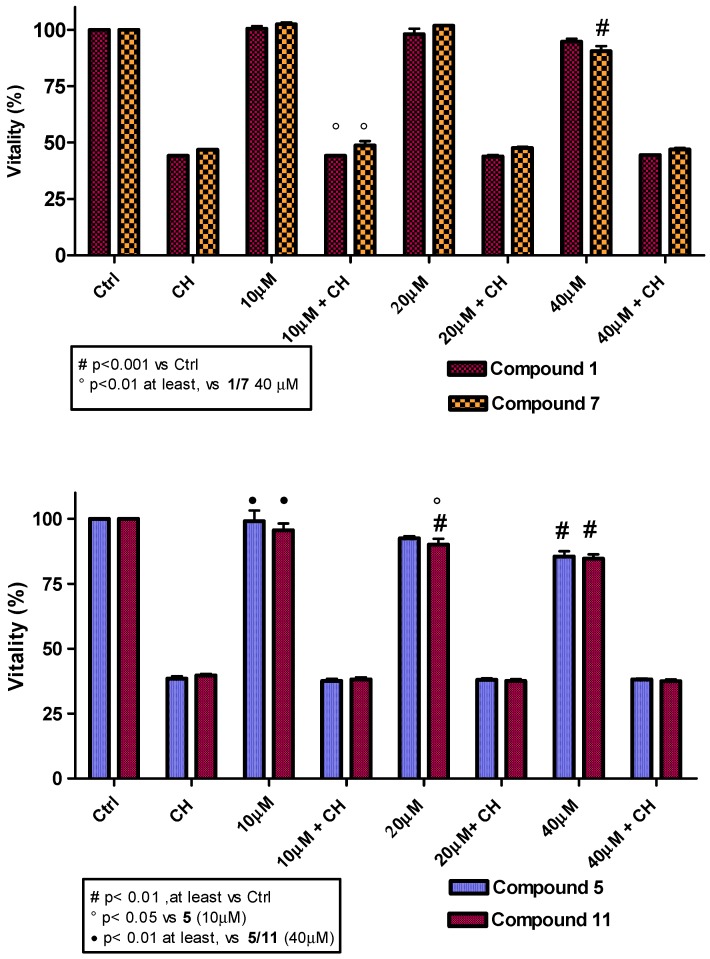

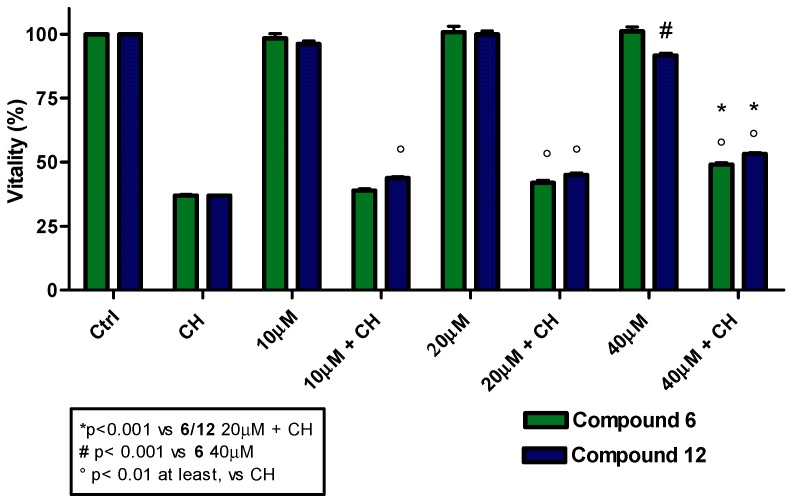
3-(4,5-dimethylthiazol-2-yl)-2,5-diphenyltetrazolium bromide (MTT) spectrophotometric assay of compounds **1**, **5**, **6**, **7**, **11**, **12** at 10 to 40 µM concentration in L6 cells in presence or absence of cumene hydroperoxide (CH, 27.5 µM). Data are reported as mean values ± SD of each compound tested in quintuplicate. Statistical analysis performed with one-way analysis of variance (ANOVA) and Bonferroni post-test. The analysis was carried out using the Prism 4 statistics program. Differences were considered significant at *p* < 0.05.

**Figure 5:**
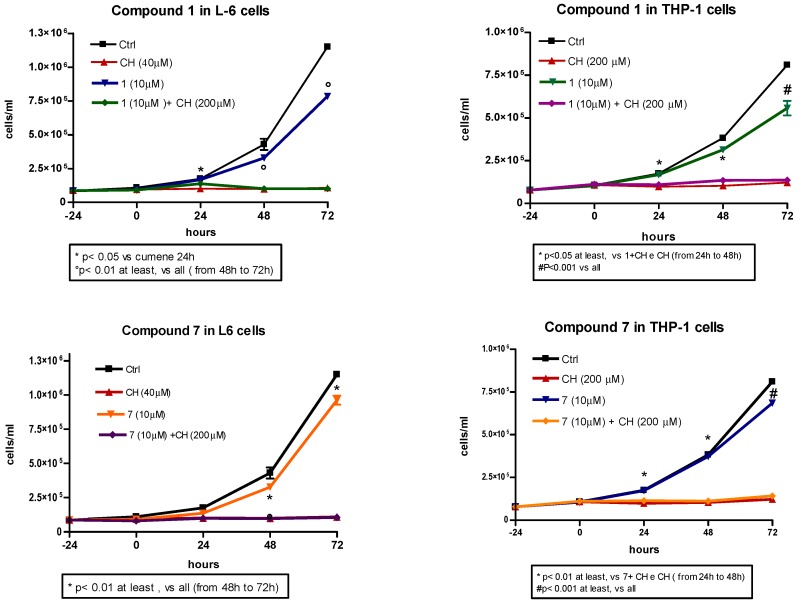
Effect of compound **1** and its ester **7** (10 µM) on the proliferation of L6 and THP-1 cells in presence or absence of cumene hydroperoxide (CH, 40 µM in L6 and 200 µM in THP-1 cells). Cell counting was done with a Neubauer chamber. Data are reported as mean values ± SD of each compound tested in duplicate. Statistical analysis performed with one-way ANOVA and Bonferroni post-test.

Considering the good antioxidant activity observed for compounds **5**, **6** and their corresponding trifluoroacetate derivatives **11** and **12**, it was essential to check the potential toxicity on cell lines and/or their effect on proliferation for cosmetic, food and pharmaceutical applications. Furthermore, based on the unexpected cellular antioxidant activity of compounds **1** and **7**, these two compounds were also included in the analyses. Employing the MTT colorimetric assay, toxicity curves were constructed using L6 myoblasts and a range of antioxidant concentrations from 10 to 40 µM (Figure 4). The results revealed a low toxicity for all compounds analyzed, with cell vitality barely diminished at concentrations higher than 20 µM. The comparable behavior of the trifluoroacetate esters and their respective alcoholic precursors suggests that, at the concentrations used, trifluoroacetic acid produced by hydrolysis does not cause any remarkable effects in the cells. Cell proliferation experiments were performed in both cell lines (L6 and THP-1). The results demonstrated a low interference in cell proliferation by all antioxidants under study, either in the presence or absence of cumene hydroperoxide. As a representative example, the cell performances for compound **1** and its corresponding trifluoroacetate derivative **7** are shown in Figure 5. Homovanillyl alcohol **5** showed a remarkable peculiarity; it promoted L6 myoblast proliferation but inhibited THP-1 tumor cell growth (Figures SI1 and SI2).

## 3. Materials and Methods

### 3.1. Chemicals

All chemicals (analytical grade), silica gel 60 F254 plates, and silica gel 60 were purchased from Sigma-Aldrich (Milan, Italy). 2′,7′-Dichlorodihydrofluorescein diacetate (DCFH_2_-DA) was supplied by Molecular Probes (Eugene, OR, USA). Fresh pure hydroxytyrosol was synthetized in our laboratory as previously reported [37]. L6 myoblasts from rat skeletal muscle and THP-1 human leukemic monocytes were purchased from the American Type Culture Collection (Rockville, MD, USA). DMEM (Dulbecco’s modified Eagle’s medium), RPMI 1640, streptomycin (100 mg/mL), penicillin (100 U/mL), d-glucose, and sterile plasticware for cell culture were from Falcon (San Diego, CA, USA). Fetal bovine serum was from GIBCO (Grand Island, NY, USA).

### 3.2. Instruments

^1^H, ^13^C, and ^19^F NMR spectra were recorded in deuterated solvents (CDCl_3_, CD_3_OD 99.8% in deuterium) using a 400 MHz nuclear resonance spectrometer (Bruker, Munich, Germany). All chemical shifts were expressed in parts per million (δ scale) and referenced to either the residual protons or carbon of the solvent. Coupling constants, *J*, were reported in Hz. GC-MS analyses were carried out on a Shimadzu VG 70/250S apparatus equipped with a Supelco SLB™ column (30 m, 0.25 mm and 0.25 μm film thickness). The analyses were performed using an isothermal temperature profile of 100 °C for 2 min, followed by a 10 °C/min temperature gradient until 280 °C for 15 min. The injector temperature was 280 °C. The fluorometric analyses were performed using a VICTOR 3V Perkin Elmer spectrofluorometer (Milan, Italy).

### 3.3. Synthesis of Phenethyl Trifluoroacetate Esters: General Procedure

Phenethyl alcohol (0.5 mmol) was dissolved in trifluoroacetic acid at room temperature (2.0 mL). The solution was kept under stirring at T = 70 °C. The reaction was monitored by thin layer chromatography (eluent: hexane/ethyl acetate = 70/30), and after 2 h, the substrate disappeared. Then, a saturated solution of NaHCO_3_ was added until neutral pH. After extraction with ethyl acetate (3 × 10 mL), the reunited organic phases were washed with a saturated solution of NaCl (2.0 mL), dried over Na_2_SO_4_, and filtered. Finally, the solvent was evaporated by distillation under reduced pressure. The oily residue was purified by silica gel chromatographic column using hexane/ethyl acetate = 70/30 as eluent and characterized by NMR and GC-MS analysis.

*Phenethyl trifluoroacetate* **7** [38]. Yield: 98%; yellow oil; ^1^H-NMR (400 MHz, CDCl_3_) δ: 7.40–7.26 (5H, m, Ph-H), 4.58 (2H, t, *J* = 8.0 Hz, OCH_2_), 3.09 (2H, t, *J* = 8.0 Hz, CH_2_); ^13^C-NMR (100 MHz, CDCl_3_) δ: 156.9 (*J*_C-F_ = 42.0 Hz), 135.7, 128.4, 128.3, 126.6, 114.0 (*J*_C-F_ = 284.0 Hz), 67.8, 34.1; ^19^F-NMR (376 MHz, CDCl_3_) δ: −75.11; GC-MS: 218 (M^+^), 120, 107, 91, 77, 69, 51.

*2-Hydroxyphenethyl trifluoroacetate* **8**. Yield: 96%; yellow oil; ^1^H-NMR (400 MHz, CDCl_3_) δ: 7.19–7.15 (2H, m, Ph-H), 6.94–6.91 (1H, t, *J* = 8.0 Hz, Ph-H), 6.78 (1H, d, *J* = 8.0 Hz, Ph-H), 4.61 (2H, t, *J* = 8.0 Hz, OCH_2_), 3.11 (2H, t, *J* = 8.0 Hz, CH_2_); ^13^C-NMR (100 MHz, CDCl_3_) δ: 156.9 (*J*_C-F_ = 42.0 Hz), 153.3, 131.0, 127.9, 122.1, 120.5, 116,4, 114.0 (*J*_C-F_ = 284.0 Hz), 66.9, 28.4; ^19^F-NMR (376 MHz, CDCl_3_) δ: −75.10. GC-MS: 234 (M^+^), 120, 107, 91, 77, 69, 51.

*3-Hydroxyphenethyl trifluoroacetate* **9**. Yield: 98%; yellow oil; ^1^H-NMR (400 MHz, CDCl_3_) δ: 7.22 (2H, t, *J* = 8.0 Hz, Ph-H), 6.83–6.74 (3H, m, Ph-H), 4.54 (2H, t, *J* = 8.0 Hz, OCH_2_), 3.01 (2H, t, *J* = 8.0 Hz, CH_2_); ^13^C-NMR (100 MHz, CDCl_3_) δ: 157.0 (*J*_C-F_ = 42.0 Hz), 156.6, 137.8, 129.8, 120.5, 115.8, 114.7, 114.0 (*J*_C-F_ = 284.0 Hz), 68.3, 34.4; ^19^F-NMR (376 MHz, CDCl_3_) δ: −75.09; GC-MS (M^+^): 234 (M^+^), 120, 107, 91, 77, 69, 51.

*4-Hydroxyphenethyl trifluoroacetate (tyrosol trifluoroacetate)* **10**. Yield: 98%; yellow oil; ^1^H-NMR (400 MHz, CDCl_3_) δ: 7.11 (2H, t, *J* = 8.0 Hz, Ph-H), 6.82 (2H, d, *J* = 8.0 Hz, Ph-H), 4.52 (2H, t, *J* = 4.0 Hz, OCH_2_), 3.00 (3H, t, *J* = 4.0 Hz, CH_2_); ^13^C-NMR (100 MHz, CDCl_3_) δ: 157.0 (*J*_C-F_ = 42.0 Hz), 154.1, 129.6, 127.9, 115.1, 114.0 (*J*_C-F_ = 284.0 Hz), 68.1, 33.2; ^19^F-NMR (376 MHz, CDCl_3_) δ: −75.10; GC-MS: 234 (M^+^), 107, 91, 77, 69, 51.

*4-Hydroxy-3-methoxyphenethyl trifluoroacetate* **11**. Yield: 88%; yellow oil; ^1^H-NMR (400 MHz, CDCl_3_) δ: 6.89 (1H, d, *J* = 8.0 Hz, Ph-H), 6.73–6.70 (2H, m, Ph-H), 4.53 (2H, t, *J* = 8.0 Hz, OCH_2_), 3.90 (3H, s, OCH_3_), 3.00 (2H, t, *J* = 8.0 Hz, CH_2_); ^13^C-NMR (100 MHz, CDCl_3_) δ: 156.9 (*J*_C-F_ = 42.0 Hz), 146.6, 144.7, 128.0, 121.6, 114.6, 114.0 (*J*_C-F_ = 284.0 Hz), 111.4, 68.2, 55.8, 34.3; ^19^F-NMR (376 MHz, CDCl_3_) δ: −75.10. GC-MS: 264 (M^+^), 150, 137, 122, 107, 91, 77, 69, 51.

*3,4-Dihydroxyphenethyl trifluoroacetate (hydroxytyrosol trifluoroacetate)* **12**. Yield. 85%; yellow oil; ^1^H-NMR (400 MHz, CD_3_COCD_3_) δ: 6.83 (1H, d, *J* = 8.0 Hz, Ph-H), 6.78–6.65 (2H, m, Ph-H), 4.49 (2H, t, *J* = 8.0 Hz, OCH_2_), 2.93 (2H, t, *J* = 8.0 Hz, CH_2_); ^13^C-NMR (100 MHz, CD_3_COCD_3_) δ: 157.0 (*J*_C-F_ = 42.0 Hz), 143.8, 142.6, 129.1, 121.4, 115.8, 115.6, 114.0 (*J*_C-F_ = 284.0 Hz), 68.5, 33.9; ^19^F-NMR (376 MHz, CD_3_COCD_3_) δ: −75.09; GC-MS: 250 (M^+^), 136, 123, 107, 91, 77, 69, 51.

### 3.4. Measurement of Partition Coefficient (logP)

The logP value of all compounds was determined as reported in the literature by UV-Vis spectrophotometry [35]. The partition coefficient (logP) was calculated according to the following relationship: logP = logA_x_/(A_0_ − A_x_). A_0_ was the maximum of absorbance of a solution 0.15 mM of each compound dissolved in 1-octanol; A_x_ is the maximum of absorbance of the organic solution containing the compound after separation from the phosphate buffer (0.1 M, pH 7.4) solution. A solution of 1-octanol saturated with the buffer was used as a blank. Experimental logP values are reported in Table 1.

### 3.5. ABTS Assay

The antioxidant capacity of compounds **1**–**12** was measured according to the method of Pellegrini et al. [39] but working at room temperature [8,9]. 6-Hydroxy-2,5,7,8-tetramethylchroman-2-carboxylic acid (Trolox) was used as reference. The analyses were carried out in ethanol (0.2% of water) at room temperature and the absorbance was measured at λ = 734 nm. Each sample (1.0 mL) of ABTS^+•^ was obtained from the aqueous mother solution by dilution with ethanol to an absorbance of 0.70 ± 0.20, and 10 μL of the ethanol solution of the antioxidant was added. All compounds were analyzed at four different final concentrations ranging from 1 to 10 μM. The absorbance was measured after 2 min. Four measurements were recorded for each concentration. Each time blanks of pure solvent were run. The standard deviations of data were all below 5%. The dose-response curves were expressed as the percentage of absorbance decrease (ABTS inhibition expressed as percentage) against the amount of antioxidant concentration. Linear regression was conducted using GraphPad Prism 4.01 (2004) software. The antioxidant capacities were extrapolated at 10 μM concentration and reported as Trolox Equivalent Antioxidant Capacity (TEAC), defined as the concentration (mmol/L) of Trolox having the antioxidant capacity equivalent to that of a 1.0 mmol/L solution of the substance under investigation. Results are expressed as mean ± standard deviation. Statistical analyses were performed using the Student’s *t* test. The level of significance was *p* < 0.05 for all data.

### 3.6. DPPH Assay

DPPH assay was performed following the method described by Brand-Williams et al. [40] with some modifications. A 75 μM solution of DPPH in methanol was prepared. Samples were diluted and analyzed at three different final concentrations ranging from 1 to 10 μM. A 50 μL volume of each sample solution was added to 0.950 mL of DPPH solution and left in the dark. After 30 min, the absorbance of samples was measured at λ = 517 nm. For the blank, 50 μL of pure ethanol was used. Four measurements were recorded for each sample. Data collected showed a standard deviation below 5%. Percentages of DPPH inhibition versus antioxidant concentrations were plotted. Linear regressions were conducted using GraphPad Prism 4 software. IC_50_ values were extrapolated from each graph as the concentration of sample that decreases by 50% DPPH radical absorbance. Statistical analyses were performed by the Student’s *t* test and one-way analysis of variance (ANOVA). Lastly, anti-radical activity (ARA), as the inverse of IC_50_, was also calculated.

### 3.7. Cells in Culture

L6 myoblasts were seeded in 75-cm^2^ tissue culture flasks and grown in DMEM containing 4.5 g/L glucose, supplemented with 10% heat-inactivated fetal bovine serum, 100 µg/mL streptomycin, and 100 U/mL penicillin, in an atmosphere of 5% CO_2_ at 37 °C. Cells reached confluency after five days (approximately 6.0 × 10^6^ cells/flask) and were kept in culture as myoblasts by continuous passages at preconfluent stages as previously reported [41]. THP-1 human leukemic monocytes were grown in suspension in RPMI 1640 medium with 10% fetal bovine serum, 100 µg/mL streptomycin, and 100 U/mL penicillin, in a humidified atmosphere with 5% CO_2_ at 37 °C [42]. THP-1 monocytes were passaged twice a week by 1:4 dilutions and re-seeded; cells from passages 7–23 were used for the experiments.

### 3.8. Intracellular ROS Determination

The antioxidant activity of each compound was measured by a standard intracellular fluorescent probe (DCF) assay [8,9,41,42] using L6 myoblasts and THP-1 monocytes exposed to oxidative stress by the addition of cumene hydroperoxide, a radical generator. Fluorescence was measured over a 10-min period, and the ability of the compounds to eliminate the increased ROS production was determined. For L6 myoblasts, growing attached to the bottom of the flask, the medium was discarded and cells were washed twice with 5 mL phosphate buffered saline (PBS) containing 5.0 mM glucose (PBS-glucose) at 37 °C. Cells were gently scraped off with PBS-glucose at 37 °C and centrifuged at 1200 rpm for 5 min (approximately 100× *g*). The supernatant was discarded and the pellet resuspended in PBS-glucose. Incubation with the probe DCFH_2_-DA at a final concentration of 10 µM (from a stock solution of 10 mM in DMSO) was carried out for 30 min at 37 °C in the dark. At the end of the incubation, cells were washed twice, centrifuged at 1200 rpm for 5 min, and the final cell pellet was resuspended in PBS-glucose. Before the experiment was initiated, cells were allowed to recover for 1 h at room temperature in the dark. The THP-1 monocytes, growing in suspension, were centrifuged at 1200 rpm for 10 min. The supernatant was discarded, and cells were washed twice with 5 mL PBS-glucose at 37 °C to remove the serum. The pellet was successively resuspended in PBS-glucose, and the probe DCFH_2_-DA was added at 10 µM final concentration. From that point onwards, the protocols for the two cell types were identical. Intracellular fluorescence was measured at 37 °C using a Perkin Elmer VICTOR 3V spectrofluorometer (excitation at λ = 498 nm and emission at λ = 530 nm). Cumene hydroperoxide in DMSO was used as the radical generator (200 μM final concentration); DMSO did not affect the fluorescence signal at the concentrations used. Cells were incubated with compounds at the final designated concentration for 10 min at 37 °C before addition of the radical generator. The decrease in the intracellular DCF fluorescence relative to the fluorescence change induced by 200 μM cumene hydroperoxide alone (100%), reported as ΔF/10 min, determined the antioxidant activities. Data are reported as the mean ± standard deviation of at least four different experiments carried out in triplicate. One-way ANOVA test (non-parametric) and Bonferroni post-test (comparing all pairs of columns) were used for the statistical analyses.

### 3.9. MTT Assay

The 3-(4,5-dimethylthiazol-2-yl)-2,5-diphenyltetrazolium bromide (MTT) spectrophotometric assay assessed proliferation/cytotoxicity. Compounds **1**, **5**–**7**, **11**, and **12** (1.0 mM) were tested and diluted in either DMEM or RPMI, supplemented as detailed above for the two cell types, to final concentrations of 10, 20, and 40 μM. L6 cells treated with and without cumene hydroperoxide (27.5 µM) [41] were seeded at a density of 10,000/well in 200 μL of complete medium in a 96-well plate. After 24 h, the DMEM was discarded and 100 μL of the test solutions was added. Following 24 h of incubation, 10 μL of MTT solution (5 mg/mL) was added to each well and incubated for a further 4 h. Lysis buffer was prepared by dissolving 40% (*w*/*v*) sodium dodecyl sulfate (SDS) in deionized water, mixing an equal volume of *N*,*N*-dimethylformamide with the SDS solution, and adjusting the pH to 4.7 [42]. After incubation with MTT, 100 μL of lysis buffer was added to each well and the absorbance read at λ = 590 nm in a microplate reader (Perkin Elmer VICTOR 3V spectrometer). Each compound was tested in quintuplicate.

### 3.10. Proliferation Curves

L6 cells were seeded in 60 × 15 mm Petri dishes in 4 mL of the complete growth medium at a density of approximately 10^5^ cells/well. The following day, the cells were exposed to the selected compounds **1**, **5**–**7**, **11**, and **12** at a final concentration of 10 µM in the absence and presence of cumene hydroperoxide (40 µM). Cell counts using a Neubauer chamber were made every 24 h up to confluence. Each time the L6 myoblasts had to be counted, they first underwent a mild trypsinization. THP-1 cells were seeded in 24-well plates in 1 mL of the growth medium. After 24 h, the cells were exposed to 10 µM of the same compounds in the absence and presence of cumene hydroperoxide (200 µM) and incubated for an additional 24 h. Cell counts using a Neubauer chamber were made every 24 h up to confluence. The results are given as the mean ± standard deviation of two different experiments.

### 3.11. Statistical Analysis

All data obtained from cultured cells were analyzed by one-way ANOVA and Bonferroni post-test. In some cases, the Student’s *t* test was applied. Analyses were carried out using the GraphPad Prism 4.1 statistics program. Differences were considered significant at *p* < 0.05.

## 4. Conclusions

Novel trifluoroacetate esters **7**–**12** were prepared in high yield by a simple and efficient procedure from phenethyl alcohols **1**–**6** in the presence of trifluoroacetic acid. Following lipophilicity evaluation, the antioxidant activity of all compounds was tested in vitro by ABTS, DPPH assays and in cell lines by the DFC assay. Cell toxicity and proliferation were also assessed. The experimental data confirmed the direct correlation between the number of phenolic groups on the aromatic ring and the radical scavenging ability. In particular, in in vitro experiments, all trifluoroacetate esters showed a similar antioxidant activity to their corresponding alcoholic precursor, while in cellular experiments, they demonstrated a weakly enhanced performance due to their higher lipophilicity. The rationale for the good antioxidant activity of phenethyl alcohol **1** and its trifluoroacetate ester **7** in the cellular environment lies beyond these considerations and may only be explained in the context of a more general analysis of the metabolic activity of this class of compounds. Specifically, their effectiveness in the cellular environment could be due to the attachment of the hydroxyl groups by cytochrome P450, as previously reported for other molecules [43]. This interesting concept will be the subject of future work in our laboratories.

## Figures and Tables

**Table 1:** LogP values of compounds **1**–**12**.

Compound	LogP ± SD ^a^
**1**	1.18 ± 0.08
**2**	0.72 ± 0.07
**3**	0.66 ± 0.06
**4**	0.69 ± 0.05
**5**	0.46 ± 0.02
**6**	0.10 ± 0.01
**7**	2.38 ± 0.18
**8**	2.05 ± 0.16
**9**	1.96 ± 0.14
**10**	1.98 ± 0.14
**11**	1.90 ± 0.12
**12**	1.62 ± 0.12

^a^ Mean of triplicate determinations.

## Supplementary Materials

The following are available online. Table SI1. DPPH assay of phenethyl alcohols **1**–**6** and trifluoroacetyl esters **7**–**12**. Table SI2. DCF assay: determination of intracellular ROS after stimulation with cumene hydroperoxide (CH) in presence of phenethyl alcohols **1**–**6** and their trifluoroacetyl esters **7**–**12** at 10 µM concentration on both cell lines L6 and THP-1. Data are reported as mean values ± SD of five experiments. Figure SI1. Effect of **5** and **11** (10 µM) on the proliferation of L6 and THP-1 cells in the absence and presence of cumene hydroperoxide (CH: 40 µM in L6 and 200 µM in THP-1 cells, respectively). Cell counting was done with a Neubauer Chamber. Figure SI2. Effect of **6** and **12** (10 µM) on the proliferation of L6 and THP-1 cells in the absence and presence of cumene hydroperoxide (CH: 40 µM in L6 and 200 µM in THP-1 cells, respectively). Cell counting was done with a Neubauer Chamber.

## References

[1] Lobo V., Patil A., Phatak A., Chandra N. (2010). Free radicals, antioxidants and functional foods: Impact on human health. Pharmacogn. Rev..

[2] Pollio A., Zarrelli A., Romanucci V., Di Mauro A., Barra F., Pinto G., Crescenzi E., Roscetto E., Palumbo G. (2016). Polyphenolic profile and targeted bioactivity of methanolic extracts from Mediterranean ethnomedicinal plants on human cancer cell lines. Molecules.

[3] D’Abrosca B., DellaGreca M., Fiorentino A., Monaco P., Zarrelli A. (2004). Low molecular weight phenols from the bioactive aqueous fraction of *Cestrum parqui*. J. Agric. Food Chem..

[4] Saladino R., Crestini C., Bernini R., Mincione E., Ciafrino R. (1995). A new and efficient synthesis of 8-hydroxypurine derivatives by dimethyldioxirane oxidation. Tetrahedron Lett..

[5] Saladino R., Bernini R., Crestini C., Mincione E., Bergamini A., Marini S., Palamara A.T. (1995). Studies on the chemistry of pyrimidine derivatives with dimethyldioxirane: Synthesis, cytotoxic effects and antiviral activity of new 5,6-oxiranyl-5,5-dihydro and 5-hydroxy-5,6-dihydro-6-substitued uracil derivatives and pyrimidine nucleosides. Tetrahedron.

[6] Shibutami S., Takeshita M., Grollman A.P. (1991). Insertion of specific bases during DNA synthesis past the oxidation-damaged base 8-oxodG. Nature.

[7] Bouallagui Z., Bouaziz M., Lassoued S., Engasser J.M., Ghoul M., Sayadi S. (2011). Hydroxytyrosol acyl esters: Biosynthesis and activities. Appl. Biochem. Biotechnol..

[8] Tofani D., Balducci V., Gasperi T., Incerpi S., Gambacorta A. (2010). Fatty acid hydroxytyrosyl esters: Structure/antioxidant activity relationship by ABTS and in cell-culture DCF assays. J. Agric. Food Chem..

[9] Bernini R., Crisante F., Barontini M., Tofani D., Balducci V., Gambacorta A. (2012). Synthesis and structure/antioxidant activity relationship of novel catecholic antioxidant structural analogues to hydroxytyrosol and its lipophilic esters. J. Agric. Food Chem..

[10] Bernini R., Carastro I., Palmini G., Tanini A., Zonefrati R., Pinelli P., Brandi M.L., Romani A. (2017). Lipophilization of hydroxytyrosol-enriched fractions from *Olea europaea* L. by-products and evaluation of the in vitro effects on a model of colorectal cancer cells. J. Agric. Food Chem..

[11] Bernini R., Gilardini Montani M.S., Merendino N., Romani A., Velotti F. (2015). Hydroxytyrosol-derived compounds: A basis for the creation of new pharmacological agents for cancer prevention and therapy. J. Med. Chem..

[12] Madrona A., Pereira-Caro G., Mateos R., Rodriguez G., Trujillo M., Fernadez-Bolanos J., Espartero J.L. (2009). Synthesis of hydroxytyrosyl alkyl ethers from olive oil waste waters. Molecules.

[13] Gerebtzoff G., Li-Blatter X., Fischer H., Frentzel A., Seelig A. (2004). Halogenation of drugs enhances membrane binding and permeation. Chembiochem.

[14] Ismail F.M.D. (2002). Important fluorinated drugs in experimental and clinical use. J. Fluor. Chem..

[15] Bovicelli P., Mincione E., Antonioletti R., Bernini R., Colombari M. (2001). Selective halogenation of aromatics by dimethyldioxirane and halogen ions. Synth. Commun..

[16] Bovicelli P., Bernini R., Antonioletti R., Mincione E. (2002). Selective halogenation of flavanones. Tetrahedron Lett..

[17] Bernini R., Pasqualetti M., Provenzano G., Tempesta S. (2015). Ecofriendly synthesis of halogenated flavonoids and evaluation of their antifungal activity. New J. Chem..

[18] Strunekà A., Patočka J., Connet P. (2004). Fluorine in medicine. J. Appl. Biomed..

[19] Zaldini Hernandes M., Cavalcanti S.M.T., Moreira D.R.M., Filgueira de Azevedo Junior W., Leite A.C.L. (2010). Halogen atoms in the modern medicinal chemistry: Hints for the drug design. Curr. Drug Targets.

[20] Barontini M., Bernini R., Carastro I., Gentili P., Romani A. (2014). Synthesis and DPPH radical scavenging activity of novel compounds obtained from tyrosol and cinnamic acid derivatives. New J. Chem..

[21] Bernini R., Crisante F., D’Acunzo F., Gentili P., Ussia E. (2016). Oxidative cleavage of 1-aryl-isochroman derivatives by the *Trametes villosa* laccase/1-hydroxybenzotroazole system. New J. Chem..

[22] Loizo M., Bonesi M., Di Lecce G., Boselli E., Tundis R., Pugliese A., Menichini F., Frega N.G. (2013). Phenolics, aroma profile, and in vitro antioxidant activity of Italian dessert passito wine from Saracena (Italy). J. Food Sci..

[23] Cosmeticinfo.org. http://www.cosmeticsinfo.org/ingredient/phenethyl-alcohol-0.

[24] U.S. Food and Drug Administration. https://www.fda.gov/Food/IngredientsPackagingLabeling/GRAS/default.htm.

[25] Zhu Y.J., Zhou H.T., Hu Y.H., Tang J.Y., Su M.X., Guo Y.J., Chen Q.X., Liu B. (2011). Antityrosinase and antimicrobial activities of 2-phenylethanol, 2-phenylacetaldehyde and 2-phenylacetic acid. Food Chem..

[26] Müller W.E.G., Falke D., Heicke B., Zahn R.K. (1973). Biological activity of 2-phenylethanol and its derivatives VIII. Influence on Herpes virus DNA-synthesis in rabbit kidney cells. Arch. Virol..

[27] Brossmer R., Bohn B., Schlicker H. (1973). Influence of 2-phenylethanol and 1,1′-dimethylphenyl-ethanol on metabolic activity and cell membrane function in Ehrlich ascites tumour cells. FEBS Lett..

[28] Romani A., Pinelli P., Ieri F., Bernini R. (2016). Sustainability, innovation and green chemistry in the production and valorization of phenolic extracts from *Olea europaea* L.. Sustainability.

[29] Bernini R., Merendino N., Romani A., Velotti F. (2013). Naturally occurring hydroxytyrosol: Synthesis and anticancer potential. Curr. Med. Chem..

[30] Bernini R., Cacchi S., Fabrizi G., Filisti E. (2008). 2-Arylhydroxytyrosol derivatives via Suzuki-Miyaura cross-coupling. Org. Lett..

[31] Bernini R., Crisante F., Merendino N., Molinari R., Soldatelli M.C., Velotti F. (2011). Synthesis of a novel ester of hydroxytyrosol and alpha-lipoic acid exhibiting an antiproliferative effect on human colon cancer HT-29 cells. Eur. J. Med. Chem..

[32] Fortunati E., Luzi F., Dugo L., Fanali C., Tripodo G., Santi L., Kenny J.M., Torre L., Bernini R. (2017). Hydroxytyrosol as active ingredient in poly(vinyl alcohol) films for food packaging applications. J. Renew. Mater..

[33] Fortunati E., Luzi F., Dugo L., Fanali C., Tripodo G., Santi L., Kenny J.M., Torre L., Bernini R. (2016). Effect of hydroxytyrosol methyl carbonate on the thermal, migration and antioxidant properties of PVA based films for active food packaging. Polym. Int..

[34] Bernini R., Mincione E., Crisante F., Barontini M., Fabrizi G., Gentili P. (2007). Chemoselective and efficient carboxymethylation of the alcoholic chain of phenols by dimethyl carbonate (DMC). Tetrahedron Lett..

[35] Grasso S., Siracusa L., Spatafora C., Renis M., Tringali C. (2007). Hydroxytyrosol lipophilic analogues: Enzymatic synthesis, radical scavenging activity and DNA oxidative damage protection. Bioorg. Chem..

[36] Leone S., Fiore M., Lauro M.G., Pino S., Cornetta T., Cozzi R. (2008). Resveratrol and X rays affect gap junction intercellular communications in human glioblastoma cells. Mol. Carcinog..

[37] Bernini R., Mincione E., Barontini M., Crisante F. (2008). Convenient synthesis of hydroxytyrosol and its lipophilic derivatives from tyrosol or homovanillyl alcohol. J. Agric. Food Chem..

[38] Chen C.-T., Kuo J.-H., Pawar V.D., Munot Y.S., Weng S.-S., Ku C.-H., Liu C.-Y. (2005). Nucleophilic acyl substitutions of anhydrides with protic nucleophiles catalyzed by amphoteric, oxomolybdenum species. J. Org. Chem..

[39] Pellegrini N., Visioli F., Buratti S., Brighenti F. (2001). Direct analysis of total antioxidant activity of olive oil and studies on the influence of heating. J. Agric. Food Chem..

[40] Brand-Williams W., Cuvelier M.E., Berset C. (1995). Use of free radical method to evaluate antioxidant activity. Lebensm. Wiss. Technol..

[41] D´Arezzo S., Incerpi S., Davis F.B., Acconcia F., Marino F., Farías R.N., Davis P.J. (2004). Rapid nongenomic effects of 3,5,3′-triiodo L-thyronine on the intracellular pH of L-6 myoblasts are mediated by intracellular calcium mobilization and kinase pathways. Endocrinology.

[42] Lombardo E., Sabellico C., Hájek J., Staňková V., Filipský T., Balducci V., De Vito P., Leone S., Bavavea E.I., Proietti Silvestri I. (2013). Protection of cells against oxidative stress by nanomolar levels of hydroxyflavones indicates a new type of intracellular antioxidant mechanism. PLoS ONE.

[43] Narasimhulu S. (2010). New cytochrome P450 mechanisms: Implications for understanding molecular basis for drug toxicity at the level of the cytochrome. Expert Opin. Drug Metab. Toxicol..

