# Supplementary material for: Synthesis and Evaluation of the Antioxidant Activity of Lipophilic Phenethyl Trifluoroacetate Esters by In Vitro ABTS, DPPH and in Cell-Culture DCF Assays
Source: Molecules. 2018 Jan 19;23(1):208. doi: 10.3390/molecules23010208 (PMC6017616; doi:10.3390/molecules23010208)
Supplement: Supplementary file 1 [file molecules-23-00208-s001.pdf]

Supporting Information

Synthesis and Evaluation of the Antioxidant Activity of Lipophilic Phenethyl Trifluoroacetate Esters by In Vitro ABTS, DPPH and in Cell-Culture DCF Assays

Roberta Bernini [1,*], Maurizio Barontini [1], Valentina Cis [1], Isabella Carastro [1], Daniela Tofani [2,3,*], Rosa Anna Chiodo [2], Paolo Lupattelli [4] and Sandra Incerpi [2]

[1] Department of Agricultural and Forestry Sciences (DAFNE), University of Tuscia, Via S. Camillo de Lellis, 01100 Viterbo, Italy; barontinimaurizio@gmail.com (M.B.); r_vale87@libero.it (V.C.); isabella109@alice.it (I.C.)

[2] Department of Sciences, University Roma Tre, Viale G. Marconi 446, 00146 Rome, Italy; rosa.anna.chiodo@gmail.com (R.A.C.); sandra.incerpi@uniroma3.it (S.I.)

[3] Centro Interdipartimentale di Servizi per la Didattica della Chimica (CIDSiC), University Roma Tre, Via della Vasca Navale 79, 00146 Rome, Italy

[4] Department of Sciences, University of Basilicata, Via dell'Ateneo Lucano 10, 85100 Potenza, Italy; paolo.lupattelli@unibas.it

* Correspondence: berninir@unitus.it (R.B.); daniela.tofani@uniroma3.it (D.T.); Tel.: +39-0761-357452 (R.B.); +39-06-57333371 (D.T.)

INDEX

Table SI1 *Pag. 2*
Table SI2 *Pag. 3*
Figure SI1 *Pag. 4*
Figure SI2 *Pag. 5*

Table SI1. DPPH assay of phenethyl alcohols **1**-**6** and trifluoroacetyl esters **7**-**12**.[a]

Compound	IC_{50}	Δ_{IC}	ARA	Δ_{ARA}
**1**	167	3	0,0060	0,0002
**2**	40,4	0,3	0,0248	0,0007
**3**	33	2	0,0301	0,0009
**4**	397	5	0,0025	0,0001
**5**	0,373	0,008	2,68	0,08
**6**	0,156	0,006	6,4	0,2
**7**	1328	31	0,0008	0,00002
**8**	1031	21	0,0010	0,00003
**9**	1163	22	0,0009	0,00003
**10**	22,1	0,6	0,045	0,001
**11**	0,50	0,02	1,99	0,06
**12**	0,123	0,004	8,1	0,24
**Trolox**	0,221	0,006	4,5	0,14

[a] IC_{50} values were extrapolated from each calibration line as the concentration of sample that decreases by 50% DPPH radical absorbance. Anti-radical activity (ARA) was calculated as the inverse of IC_{50}. Statistical analyses were performed by Student's *t* test and one-way analysis of variance (ANOVA).

Table SI2. DCF assay: determination of intracellular ROS after stimulation with cumene hydroperoxide (CH) in presence of phenethyl alcohols **1-6** or their trifluoroacetyl esters **7-12** at 10 μM concentration on both cell lines L6 and THP-1. Data are reported as mean values ± SD of five experiments. Statistical analysis performed with one-way ANOVA test and Bonferroni post-test and for **4**, **6**, and **12** with Student´s *t* test.

	**L-6**			**THP-1**		
**Compounds**	**Mean**	**SD**	**N**	**Mean**	**SD**	**N**
**CH**	100	1	76	100	1	51
**1**	31	6	4	33	6	3
**2**	23	9	7	25	6	3
**3**	25	7	7	23	8	5
**4**	41	7	13	25	6	3
**5**	22	6	6	21	3	4
**6**	7	2	4	14	5	3
**7**	29	7	6	30	3	3
**8**	26	7	8	25	7	5
**9**	25	7	3	28	3	4
**10**	21	10	7	20	5	5
**11**	20	7	3	23	4	4
**12**	4	1	5	16	3	3

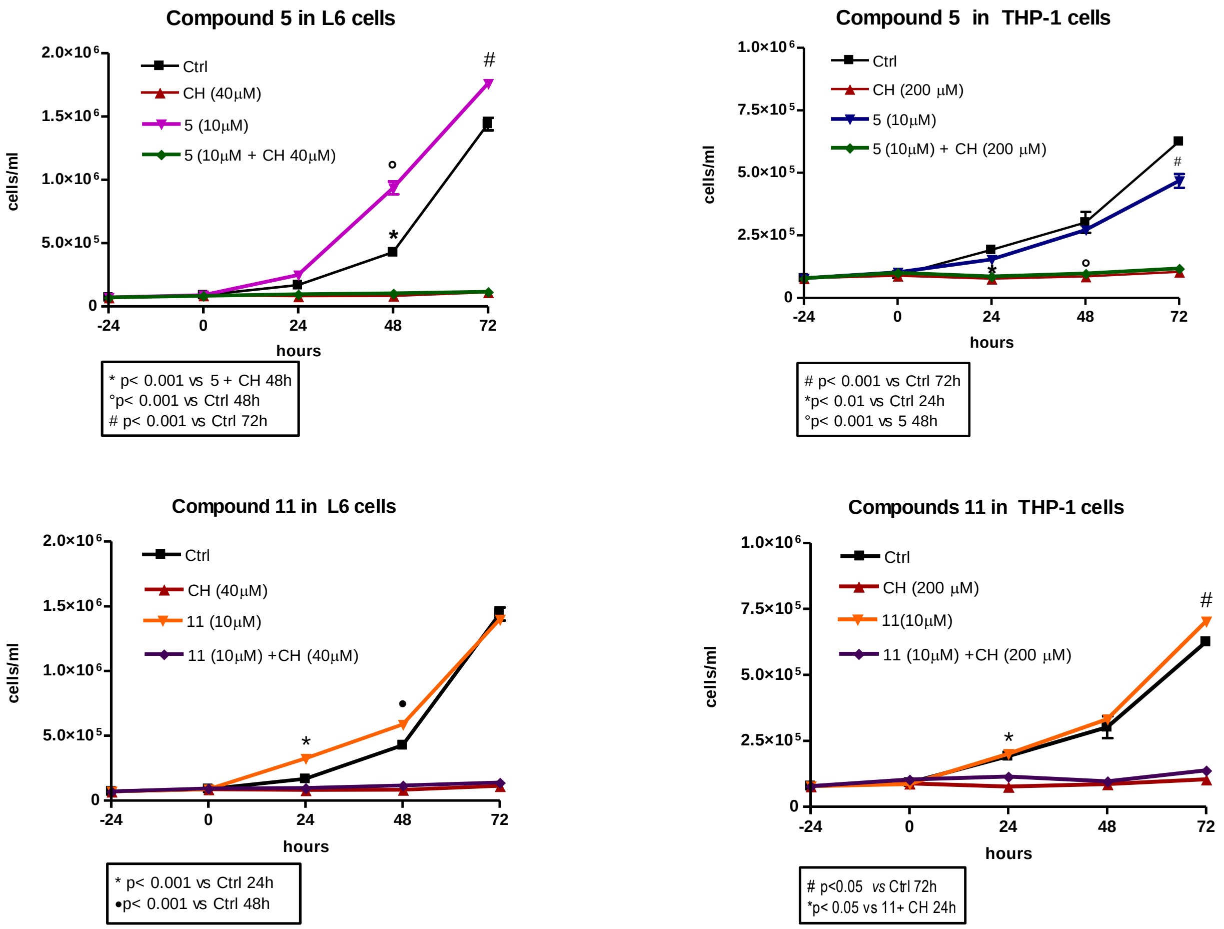

Figure SI1. Effect of **5** and **11** (10 μM) on the proliferation of L6 and THP-1 cells in the absence and presence of cumene hydroperoxide (CH: 40 μM in L6 and 200 μM in THP-1 cells, respectively). Cell counting was done with a Neubauer Chamber. Data are reported as mean values ± SD of each compound tested in duplicate. Statistical analysis performed with one-way ANOVA test and Bonferroni post-test.

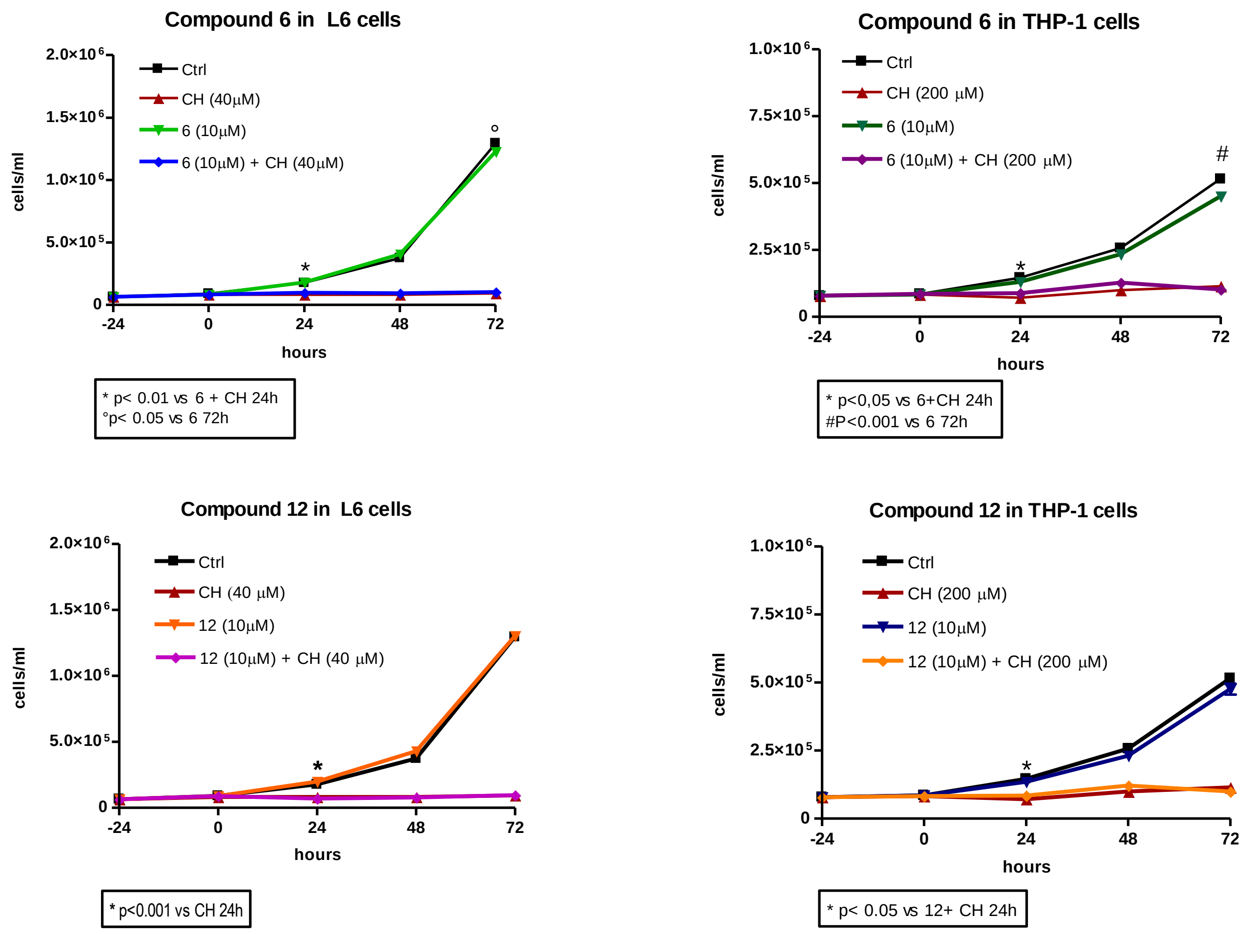

Figure SI2. Effect of **6** and **12** (10 μM) on the proliferation of L6 and THP-1 cells in the absence and presence of cumene hydroperoxide (CH: 40 μM in L6 and 200 μM in THP-1 cells, respectively). Cell counting was done with a Neubauer Chamber. Data are reported as mean values ± SD of each compound tested in duplicate. Statistical analysis performed with one-way ANOVA test and Bonferroni post-test.
